# Characterizing executive dysfunctions in patients with schizo-obsessive comorbidity: comparing schizophrenia with obsessive-compulsive disorder

**DOI:** 10.1017/S0033291726104061

**Published:** 2026-04-27

**Authors:** Min-yi Chu, Neng-jun Zhu, Shuai-biao Li, Yi Wang, Zheng-hui Yi, Yao Zhang, Qin-yu Lv, Simon S.Y. Lui, Zhen Wang, Raymond C.K. Chan

**Affiliations:** 1 Shanghai Mental Health Center, Shanghai Jiao Tong University School of Medicine, Shanghai, China; 2 School of Computer Engineering and Science, Shanghai University, Shanghai, China; 3 Neuropsychology and Applied Cognitive Neuroscience Laboratory, State Key Laboratory of Cognitive Science and Mental Health, Institute of Psychology, Chinese Academy of Sciences, Beijing, China; 4 Department of Psychology, University of Chinese Academy of Sciences, Beijing, China; 5 Department of Psychiatry, Huashan Hospital, Fudan University, School of Medicine, Shanghai, China; 6 Institute of Mental Health, Fudan University, Shanghai, China; 7 Department of Psychiatry, School of Clinical Medicine, The University of Hong Kong, Hong Kong Special Administrative Region, China

**Keywords:** executive function, machine learning, obsessive-compulsive disorder, schizo-obsessive comorbidity, schizophrenia

## Abstract

**Background:**

The term ‘schizo-obsessive comorbidity (SOC)’ is used to describe the presence of obsessive-compulsive symptoms or obsessive-compulsive disorder (OCD) in patients with schizophrenia (SOC). Recent studies have found overlapped executive dysfunctions in SCZ and OCD implicating shared pathophysiology. However, specific deficits in the components of executive function (EF) in patients with SOC remains unclear.

**Methods:**

We recruited 37 patients with SOC, 68 patients with SCZ, 70 patients with OCD, and 59 healthy controls (HCs). All participants completed a battery of measures for EF components, namely initiation, sustained attention, online updating, switching, disinhibition, and planning. Apart from traditional group-mean analysis, we applied machine learning approaches to identify the unique patterns of EF among different clinical groups.

**Results:**

The results showed that the three clinical groups could be distinguished from HCs. The feature importance analysis showed that, to classify clinical groups from HC, online updating was the core feature of SCZ patients, whereas disinhibition and online updating jointly determine classification between OCD patients and HC. In differentiating SOC from HC, online updating, planning, and disinhibition collectively served as key features. Machine learning algorithms classified SOC and OCD with acceptable performance but classified SOC and SCZ with lower performance.

**Conclusions:**

Deficits of EF are shared features among patients with SOC, SCZ, and OCD. However, the specific components of executive dysfunction in these clinical groups appeared distinct.

## Introduction

The term ‘schizo-obsessive comorbidity’ (SOC) refers to the co-occurrence of schizophrenia (SCZ) and obsessive-compulsive disorder (OCD)/obsessive-compulsive symptoms (Midou, Sanku, Lui, & Eyerman, 2025; Tezenas du Montcel, Pelissolo, Schürhoff, & Pignon, 2019). Around one-third of SCZ patients have comorbid obsessive-compulsive symptoms, and about 10–24% meet the diagnosis of OCD (Midou, Sanku, Lui, & Eyerman, 2025; Sancak & Özgen Hergül, 2022). Moreover, SCZ and OCD patients share neuropsychological deficits, which may explain such comorbidity in symptoms and diagnosis (Ricci, Martinotti, & Maina, 2025). However, it remains unclear whether SOC patients have subtle neuropsychological deficits different from SCZ and OCD.

Executive dysfunction is a candidate endophenotypic marker for SCZ and OCD (Johnsen et al., 2024; Perera et al., 2023; Yuan et al., 2025; Zartaloudi, Laws, & Bramon, 2019). Notably, SCZ patients exhibit varying extents of executive function (EF) impairments across different stages of the illness and unaffected first-degree relatives of SCZ patients (Chan, Chen, & Law, 2006; Lee et al., 2024; Mondragón-Maya, Flores-Medina, González-Sánchez, & Hernández-Echeagaray, 2023). Likewise, EF dysfunction is also a trait-related characteristic of OCD that occurs in both patients with OCD and their unaffected relatives (Bey et al., 2018; Zartaloudi, Laws, & Bramon, 2019). The shared executive dysfunctions in SCZ and OCD reflect a common pathophysiology (Dijkstra, Vermeulen, de Haan, & Schirmbeck, 2021; Friedman & Robbins, 2022). The supervisory attentional system (SAS) model views EF as a fractionated construct with specific components, including initiation, sustained attention, online updating, switching, disinhibition, and planning (Chan, Chen, & Law, 2006). Previous studies have shown that both patients with SCZ and OCD exhibit wide-ranging impairments across different components of EF (Abramovitch, De Nadai, & Geller, 2021; Berberian et al., 2019). On the other hand, preliminary evidence also suggested several ‘unique’ impairments in components of EF for each of the two disorders, such as more prominent impaired online updating for SCZ patients (Berberian et al., 2019; Chan et al., 2010), and more severely impaired switching and inhibition for OCD (Abramovitch, De Nadai, & Geller, 2021; Jalal, Chamberlain, & Sahakian, 2023).

Patients comorbid with SCZ and OCD/obsessive-compulsive symptoms have seldom been studied, using the ‘fractionated EF’ approach, and previous results are limited and inconsistent (Cunill, Huerta-Ramos, & Castells, 2013; Dijkstra, Vermeulen, de Haan, & Schirmbeck, 2021). Preliminary findings showed that patients with SOC perform worse than SCZ patients in switching (Chu et al., 2025; Schirmbeck et al., 2013) and planning (Sahoo, Grover, & Nehra, 2018) components of EF, supporting the ‘double jeopardy’ hypothesis, which posited that SCZ patients with obsessive-compulsive symptoms would have more severe clinical symptoms and cognitive deficits than patients with OCD or SCZ alone (Cunill et al., 2023; Midou, Sanku, Lui, & Eyerman, 2025; Wang et al., 2020). However, other studies reported the opposite results (Borkowska, Pilaczyñska, & Rybakowski, 2003; Lee et al., 2009), undermining such a hypothesis. A meta-analysis reported that, compared with patients with SCZ alone, patients with SOC did not show any statistically significant impairments in all components of EF (Dijkstra, Vermeulen, de Haan, & Schirmbeck, 2021). However, this meta-analysis did not utilize an OCD group and only compared the differences between the SCZ patients and the SOC patients. To our knowledge, few studies have systematically explored the specific components of EF in patients with SOC, using theory-based EF measures aligned with the fractionation of EF.

This study aimed to explore the EF profiles among patients with SOC, SCZ, OCD, and HC. To fractionate EF into six components, we adopted the same approach as in our previous works on SCZ and bipolar disorder (Chan et al., 2006; Chan, Chen, & Law, 2006; Leung et al., 2016). Moreover, apart from the traditional tests, which compared group means, we used the machine learning approach to identify the unique patterns for different clinical groups. Machine learning is a robust method to unveil complex classification patterns and identify distinct features between groups. The classification performance relies heavily on the data quality and feature selection. For example, the key components are vital for training models. Instead of pursuing the best classification performance by comparing various models, we focused on testing whether the selected components could help to train a better classifier using a particular model and how they contribute to classifications. We hypothesized that (1) the EF could effectively classify the different groups, particularly the clinical groups (SOC, SCZ, OCD) and HC and (2) the critical components of EF contributing to classifications of the different groups would be different. Specifically, online updating would chiefly contribute to classifying SCZ from other groups, whereas disinhibition and switching would be pivotal in classifying OCD from other groups, and the three components would all contribute to classifying SOC from other groups.

## Methods

### Participants

One hundred seventy-five outpatients were recruited from the Shanghai Mental Health Center according to the Diagnostic and Statistical Manual of Mental Disorders (DSM-IV) criteria (APA, 2000) comprising 37 patients with SOC, 68 with SCZ, and 70 with OCD. Participants’ medications are reported in the Supplementary Materials. Patients with SOC were recruited based on their concurrent fulfillment of the full DSM-IV diagnostic criteria for both SCZ and OCD at the time of enrollment. The inclusion criteria for clinical participants were as follows: (1) aged 18–45; (2) estimated intelligence quotient (IQ) ≥70; and (3) Han ethnicity. Exclusion criteria included a history of (1) electroconvulsive therapy in the past 3 months; (2) neurological disorder; (3) head injury with loss of consciousness for more than 30 minutes; and (4) history of substance abuse in the past 12 months.

We also recruited 59 HC from the community. The inclusion criteria for controls were the same as those for clinical participants, except that controls should not have a lifetime or family history of psychiatric disorder, as evidenced from the comprehensive diagnostic evaluation using the MINI-International Neuropsychiatric Interview (MINI) conducted by an experienced psychiatrist (ZY). We complied with the ethical standards of the relevant national and institutional committees on human experimentation and with the Helsinki Declaration of 1975, as revised in 2008. This study was approved by the Ethics Committee of the Shanghai Mental Health Center (NO. 2018‐35, 2022‐62). All participants gave written informed consent.

### Measures

#### Executive function tests

The EF tests in this study were selected based on two key criteria: (1) theoretical relevance to specific EF components or established neural correlates in prefrontal regions, and (2) demonstrated sensitivity to both quantitative and qualitative aspects of EF in healthy and clinical populations.

*The Hayling Sentence Completion Test (HSCT):* Chinese version was administered to assess semantic initiation and inhibition (Burgess & Shallice, 1996; Chan, Chen, Cheung, & Cheung, 2004). The HSCT consists of Parts A and B, each containing 15 sentences with the last word incomplete. Part A (initiation section) required participants to fill in the missing word as soon as possible, correctly. The number of correct responses was recorded as an indicator of initiation ability. Conversely, in Part B (inhibition section), participants were asked to inhibit the correct and automatic response but given a nonsense word entirely irrelevant to the content of the whole sentence. The total error numbers were used to reflect the disinhibition component.

*The Modified Six Elements Test (MSET):* The Chinese version was used to measure planning and allocation strategy ability (Chan & Manly, 2002; Wilson et al., 1996). The MSET contains three types of tasks, namely dictation, simple arithmetic, and picture naming, and each task consists of two subtasks (Parts A and B). Participants face the dual challenge of: (1) strategically allocating limited time (10 minutes) across multiple competing tasks, while (2) adhering to the critical constraint prohibiting direct transitions between analogous subtasks (e.g., cannot directly switch from Dictation-A to Dictation-B). The raw score and profile score were calculated as an index of ‘planning’. The number of effective shifts was calculated from the total shifting times minus the rule-breaking time throughout the test. We also computed the number of rule-breaking errors as an indicator of disinhibition.

*The Modified Sustained Attention to Response Task (SART)* is a computer-based task to assess sustained attention and inhibition (Chan, 2001; Robertson et al., 1997). Participants were required to press the left key of the mouse as soon as possible when the digits 1–9, except the digit ‘3’, appeared on the screen. The commission error (pressing incorrectly to digit ‘3’) and the number of correct presses were recorded, representing disinhibition and sustained attention, respectively.

*The 2-back task* (Callicott et al., 1998) and *The Letter-Number Span test (LNST)* (Chan et al., 2008; Gold et al., 1997) were used to measure the online updating. Participants were presented with four circles on the screen, and four numbers (2, 4, 6, and 8) would successively appear one at a time inside the circles. In the 2-back, participants needed to press the number key corresponding to the number that appeared two trials prior (n – 2). The Chinese version of the LNST test used in the present study required participants to arrange the mixed digits and Chinese character combinations read by the experimenter in the correct order (Chan et al., 2008). The task had eight difficulty levels (2–9 span length), each with four items. The total number of items passed in the LNS test and the 2-back accuracy were recorded.

#### Clinical assessments

The psychiatric symptoms of the SCZ and SOC groups were assessed by the Positive and Negative Syndrome Scale (PANSS) (Kay, Fiszbein, & Opler, 1987). The Yale-Brown Obsessive Compulsive Scale (Y-BOCS) was applied to the SOC and OCD groups (Goodman et al., 1989). The PANSS and Y-BOCS were administered by a qualified psychiatrist (ZY).

#### Other measures

We used the Chinese versions of the Beck Depression Inventory (BDI) (Beck, Steer, & Carbin, 1988b; Wang, Wang, & Ma, 1999) and the Beck Anxiety Inventory (BAI) (Beck, Epstein, Brown, & Steer, 1988a; Cheng et al., 2002) to measure depressive and anxiety symptoms. The Chinese version of the self-report Obsessive-Compulsive Inventory-Revised (OCI-R) was used to measure obsessive-compulsive symptoms (Foa et al., 2002; Leung et al., 2016). The estimated IQ was prorated by the short-form (arithmetic, common sense, digit span, and similarities) of the Chinese version of the Wechsler Adult Intelligence Scale-Revised (Gong, 1992).

### Statistical analyses

We recorded the scores of 10 parameters based on the set of EF tests (HSCT, SART, LNST, 2-back, MSET). Supplementary Table S1 shows the group differences in the raw scores of these parameters. We transformed the raw scores into z-standardized scores for deriving the six executive components using the fractioned EF approach (Chan, Chen, & Law, 2006; Chan et al., 2006; Leung et al., 2016): that is, (1) initiation: correct responses number in HSCT-part A; (2) sustained attention: correct press number in SART; (3) online updating: a composite score calculated by averaging the z-score of the total passed number in LNST and 2-back accuracy; (4) switching: the effective shifting number in MSET; (5) disinhibition: a composite score calculated by averaging the z-score of three parameters, including commission errors of SART, total errors number in HSCT-Part B, and number of rule-breaking errors in MSET; and (6) planning: a composite score calculated by averaging the z-score of profile score and raw score in MSET.

The Statistical Package for Social Science (SPSS) was used for data analysis. We used analysis of variances (ANOVA) to compare the standardized scores of six executive components between the groups, with partial-eta-squared calculated as the corresponding effect size to quantify the magnitude of group differences. To account for the potential confounding effects of medication on cognitive performance, we further included antipsychotic and antidepressant variables as covariates, for those specific components of executive function which showed significant post-hoc differences across the three clinical groups. The covariate analyses allowed us to clarify whether the between-group disparities would remain statistically significant after accounting for the confounding effects of psychotropic medications. We further examined the relationships between the six executive components and clinical symptoms (PANSS, Y-BOCS, BDI, BAI) using correlational analyses in each clinical group, with Bonferroni corrections applied per group to adjust for multiple comparisons. Specifically, significance thresholds were set at *p* < 0.0016 for SCZ [0.05/(5 × 6)], *p* < 0.0021 for OCD [0.05/(4 × 6)], and *p* < 0.0012 for SOC [0.05/(7 × 6)].

**Figure 1.**
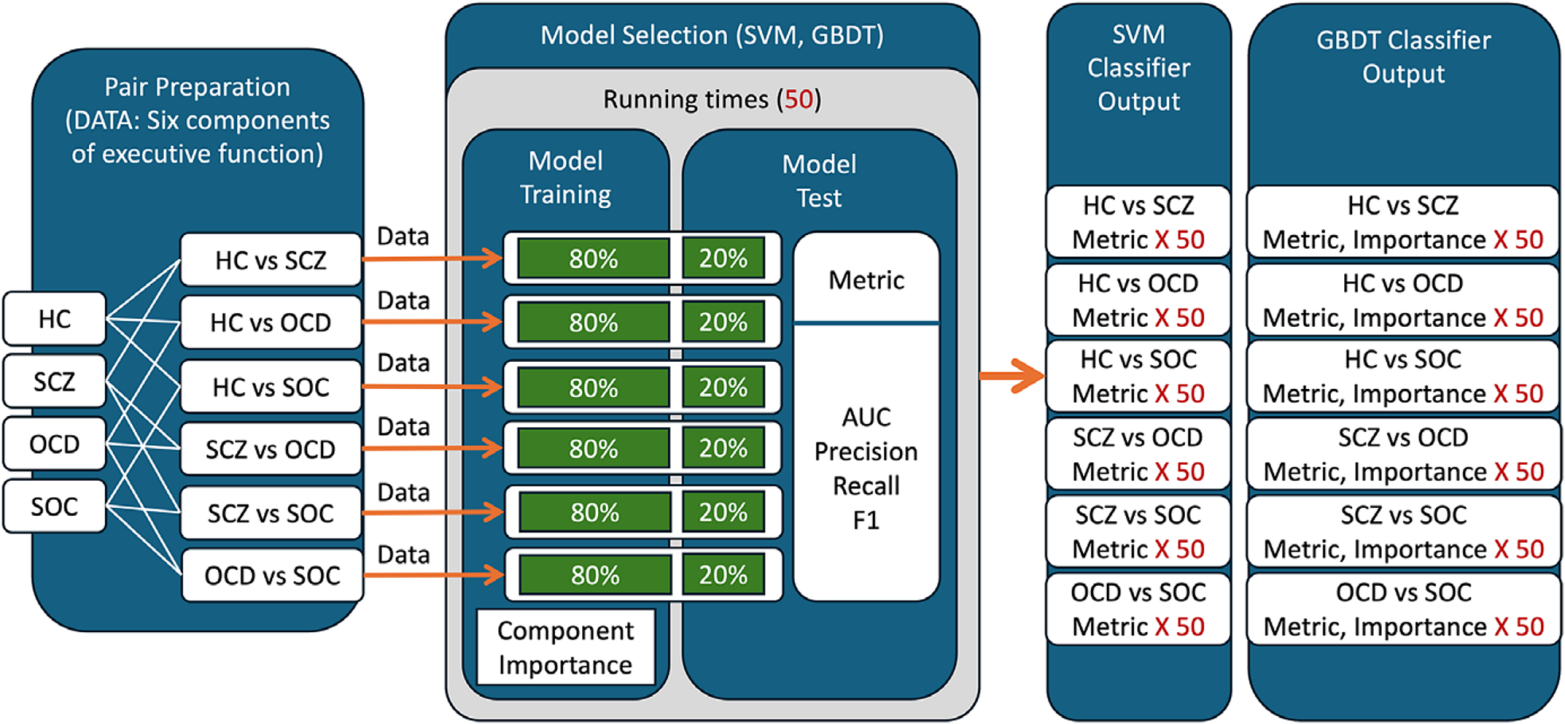
Flow chart of the machine learning approach. GMDT: Gradient Boosting Decision Tree; SVM: Support Vector Machine.

Figure 1 illustrates the key processes for the machine learning approaches we used: *(a) Model selection:* Two classifiers, the support vector machine (SVM) and the gradient boosting decision tree (GBDT), were implemented via the scikit-learn package in Python 3.7.10 with some tuned parameters (Pedregosa et al., 2011), and trained on data of the six EF components. According to their classification results, the classification performance and component importance are evaluated. *(b) Pair preparation:* Six EF components were organized by pairing two of the four groups (HC, SCZ, OCD, and SOC), with each pair forming a dataset and corresponding SVM/GBDT classifiers. *(c) Model training:* To train SVM and GBDT and to obtain the classifiers for each pair, 80% of the samples were randomly selected for training and 20% for testing. The key parameters of SVM/GBDT are listed in the Supplementary Materials. *(d) Model test:* Each pair’s data division and training were repeated 50 times independently. Performance metrics (AUC, Precision, Recall, and F1) were calculated for each classifier, with mean and standard deviation derived from 50 runs. We also used GBDT estimated feature importance, with paired *t*-tests comparing importance difference between components (significance level at 0.003 after multiple comparison correction; see the Supplementary Materials for details).

## Results

### Baseline summary

Table 1 shows the demographics of the groups with SOC, SCZ, OCD, and HC. Notably, the groups did not differ in gender (*x* ^2^ = 0.477, *p* = 0.924) and age (*F* = 1.876, *p* = 0.134). However, patients with SOC showed significantly lower education years than the OCD group (*F* = 3.171, *p* = 0.025). Compared with HC, the three clinical groups had significantly lower estimated IQ, and both SOC and SCZ groups also had lower estimated IQ than OCD groups (*F* = 10.039, *p* < 0.001). The three clinical groups did not differ in illness durations (*F* = 1.526, *p* = 0.220).

**Table 1.** Demographic information of the participants

	SCZ(*n* = 68)	OCD(*n* = 70)	SOC(*n* = 37)	HC(*n* = 59)	*F/x* ^2^*/t*	*p*	Eta^2^/Cohen’s d	post-hoc^a^
Gender (male: female)	34:34	34:36	16:21	29:30	0.477	0.924		
Age (years)	25.26 ± 5.24	27.26 ± 6.43	25.35 ± 6.42	24.81 ± 7.58	1.876	0.134	0.024	
Length of education (years)	13.93 ± 2.27	14.64 ± 2.6	13.05 ± 2.63	13.93 ± 2.75^b^	3.171	0.025	0.040	3 < 2
IQ estimates	101.93 ± 15.05	108.63 ± 13.99^c^	99.43 ± 16.17	114.1 ± 16.18	10.039	<0.001	0.117	1,3 < 2 < 4
Duration of illness (years)	4.82 ± 3.72	6.01 ± 5.36	6.21 ± 4.9		1.526	0.220	0.017	
PANSS								
Positive symptoms	16.04 ± 6.61		19.33 ± 4.9^b^		−2.626	0.01	−0.541	
Negative symptoms	17.15 ± 7.21		18.97 ± 6.26^b^		−1.283	0.202	−0.265	
General psychopathology	32.29 ± 9.29		39.78 ± 10.05^b^		−3.797	<0.001	−0.783	
total	65.49 ± 19.32		78.08 ± 18.61^b^		−3.204	0.002	−0.660	
Y-BOCS								
Obsession		11.8 ± 3.46	11.76 ± 4.35		0.056	0.955	0.011	
Compulsion		10.16 ± 4.79	10.22 ± 6.01		−0.055	0.956	−0.011	
total		21.96 ± 6.71	21.97 ± 8.29		−0.011	0.992	−0.002	
OCI_R	9.53 ± 7.33	30.77 ± 14.40^b^	31.57 ± 10.05	9.1 ± 6.95^b^	85.721	<0.001	0.530	2, 3 > 1,4
BDI	9.83 ± 8.95^d^	18.49 ± 11.31^b^	20.33 ± 11.88^b^	4.78 ± 5.73	31.33	<0.001	0.296	2, 3 > 1 > 4
BAI	7.28 ± 8.18^b^	17.37 ± 13.27	22.06 ± 12.94^b^	3.56 ± 4.34	36.609	<0.001	0.325	3 > 2 > 1 > 4

*Note:* BAI, Beck Anxiety Inventory; BDI, Beck Depression Inventory; HC, Healthy controls; OCD: Obsessive-compulsive disorder; OCI_R, Obsessive-compulsive Inventory-Revised; PANSS, Positive and Negative Syndrome Scale; SCZ, Schizophrenia; SOC, Schizo-obsessive comorbidity; Y-BOCS, Yale-Brown Obsessive Compulsive Scale.

a The numbers in the post hoc-column refer to 1, patients with Schizophrenia, 2, patients with Obsessive-compulsive disorder group; 3, patients with Schizo-obsessive comorbidity, 4, Healthy controls.

b One missing data.

c Two missing data.

d Four missing data.

Compared with patients with SCZ, patients with SOC exhibited significantly higher positive symptoms (*t* = −2.626, *p* = 0.01) and general psychopathology symptoms (*t* = −3.797, *p* < 0.001) of PANSS, but they showed no difference in negative symptoms (*t* = −1.283, *p* = 0.202). The SOC and OCD groups did not differ in the Y-BOCS obsessions subscale (*t* = 0.056, *p* = 0.955) and compulsive subscale (*t* = −0.055, *p* = 0.956). Furthermore, OCD and SOC had higher scores in OCI-R than SCZ and HC, but there was no difference between SCZ and HC (*F* = 85.721, *p* < 0.001). There were also significant differences in BDI (*F* = 31.33, *p* < 0.001) and BAI (*F* = 36.609, *p* < 0.001) scores among the four groups. Further post-hoc analysis showed that compared with HC, the three clinical groups reported higher BDI and BAI scores; both SOC and OCD patients exhibited higher BDI and BAI scores than the SCZ group, and the SOC patients also exhibited higher BAI scores than the OCD group.

### Group differences in specific components of EF

The group-means analysis showed that both the SOC and SCZ groups performed significantly worse in EF than the OCD group, affecting sustained attention, planning, and online updating. Compared with the SCZ group, SOC patients showed significantly poorer performance in planning. Furthermore, compared to HC, the three clinical groups (SCZ, SOC, and OCD) exhibited significant impairments in online updating and disinhibition (see Table S2 in the Supplementary Materials).

To address potential confounding by IQ, education, and medications, we conducted separate multivariable linear regression analyses, with each of these three confounding variables included as a covariate. The results showed that, consistent with the original findings, the inclusion of education did not alter any group differences in specific EF components. Likewise, the inclusion of IQ also did not alter the core pattern of group distinctions, though we observed the changes of results, indicating non-significant pairwise comparisons in three components, that is, the results of SCZ versus OCD in sustained attention and planning; and SOC versus OCD in online updating.

Notably, after controlling for antipsychotic dosage and antidepressant drug-class in the three clinical groups, the core pattern of between-group differences in EF components was preserved. Specifically, SOC patients performed significantly worse than OCD patients in sustained attention (*p* = 0.010) and planning (*p* = 0.028); SCZ patients exhibited significantly poorer online updating than OCD patients (*p* = 0.045). Suggestive differences were observed in planning, such as SCZ patients tended to underperform compared to OCD patients (*p* = 0.054), and SOC patients tended to underperform compared to SCZ patients (*p* = 0.063).

Correlation analysis of EF components and clinical symptoms showed that only two significant correlations were retained after Bonferroni correction: Regarding SCZ patients, initiation was negatively correlated with the BDI score (*r* = −0.4262, *p* < 0.001), suggesting that SCZ patients with more severe depression exhibited poorer initiation ability. Regarding SOC patients, disinhibition was positively correlated with PANSS negative symptoms (*r* = 0.541, *p* < 0.001), indicating that SOC patients with higher levels of negative symptoms showed more pronounced deficits in inhibition (see Table S3 in the Supplementary Materials).

### Results of machine learning

#### Classification results

**Figure 2.**
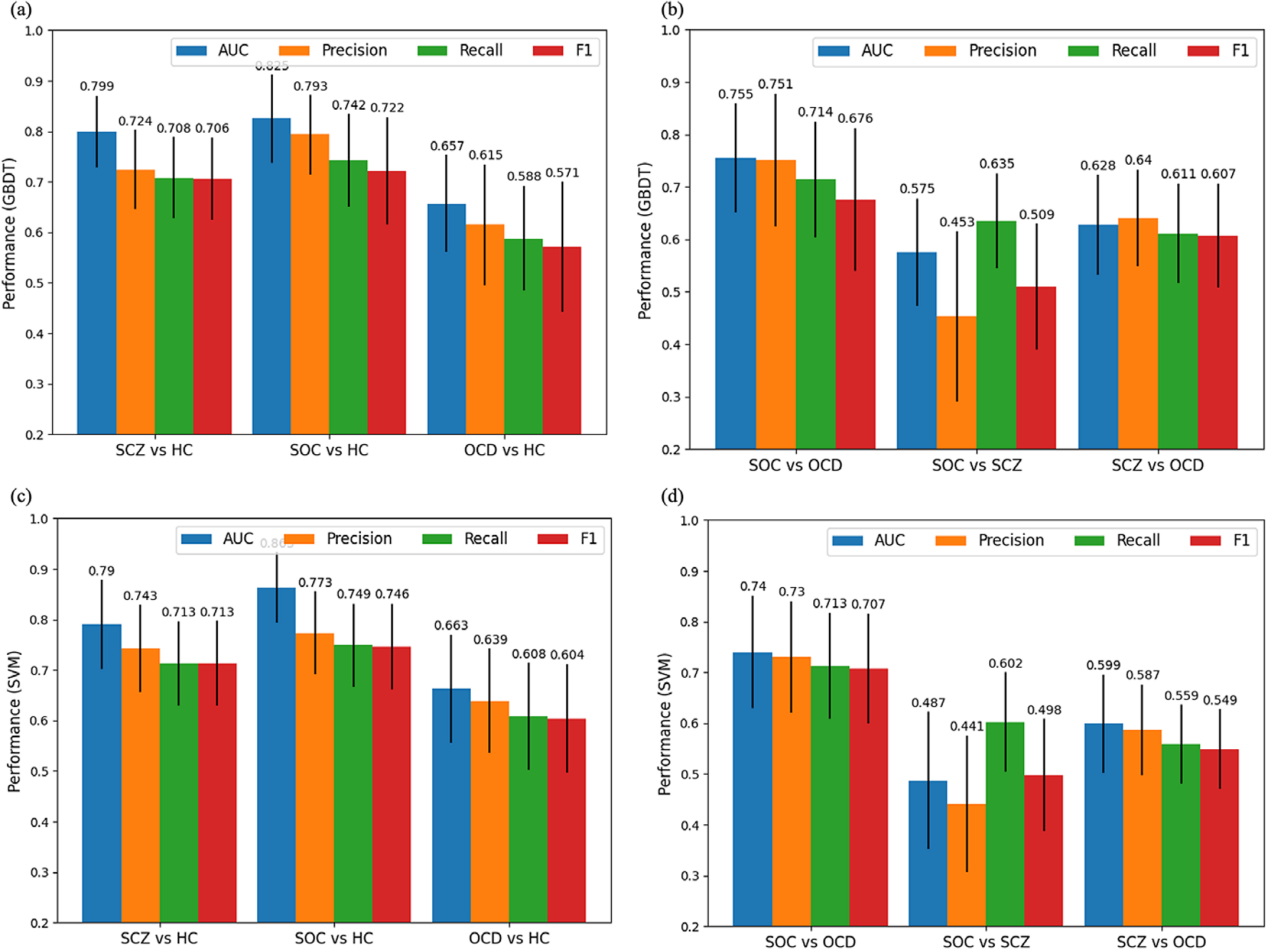
Bar chart of classification results. (a) Performance between clinical groups (SCZ, SOC, and OCD) and HC based on GBDT model; (b) Performance among clinical groups (OCD versus SOC, SCZ versus SOC, and SCZ versus OCD) based on GBDT model; (c) Performance between clinical groups (SCZ, SOC, and OCD) and HC based on SVM model; (d) Performance among clinical groups (OCD versus SOC, SCZ versus SOC, and SCZ versus OCD) based on SVM model; GMDT: Gradient Boosting Decision Tree; SVM: Support Vector Machine.

The classification performance of GBDT showed that the four metrics (i.e., AUC, Precision, Recall, and F1) achieved an acceptable performance in classifying groups in the pairs of HC and SOC, SCZ, and OCD (see Figure 2 and Table S4 in the Supplementary Materials). Specifically, the AUC, Precision, Recall, and F1 were 0.825 ± 0.087, 0.793 ± 0.078, 0.742 ± 0.092, and 0.722 ± 0.106, respectively for the HC and SOC pair; 0.799 ± 0.071, 0.724 ± 0.078, 0.708 ± 0.080, and 0.706 ± 0.081, respectively for the HC and SCZ pair; 0.657 ± 0.096, 0.615 ± 0.119, 0.588 ± 0.104, and 0.571 ± 0.129, respectively for the HC and OCD pair. The metric values between HC versus SOC, and HC versus SCZ were similar but higher than those of HC versus OCD.

When classifying SOC and OCD patients, the values of AUC, Precision, Recall, and F1 were 0.755 ± 0.104, 0.751 ± 0.126, 0.714 ± 0.110, and 0.676 ± 0.136, respectively, higher than those of SOC versus SCZ patients (i.e., 0.575 ± 0.103, 0.453 ± 0.162, 0.635 ± 0.090, and 0.509 ± 0.120 for AUC, Precision, Recall, and F1, respectively). These comparison results showed that the model trained on the data with EF could not classify SOC and SCZ patients very well. Finally, regarding the SCZ and OCD pair, the classifier could identify SCZ from OCD patients, but the results were only modest, with the AUC, Precision, Recall, and F1 of 0.628 ± 0.095, 0.640 ± 0.092, 0.611 ± 0.095, and 0.607 ± 0.099, respectively. Moreover, the SVM classifiers performed similarly to the GBDT (see Figure 2 and Table S4 in the Supplementary Materials).

#### Results of feature importance

**Figure 3.**
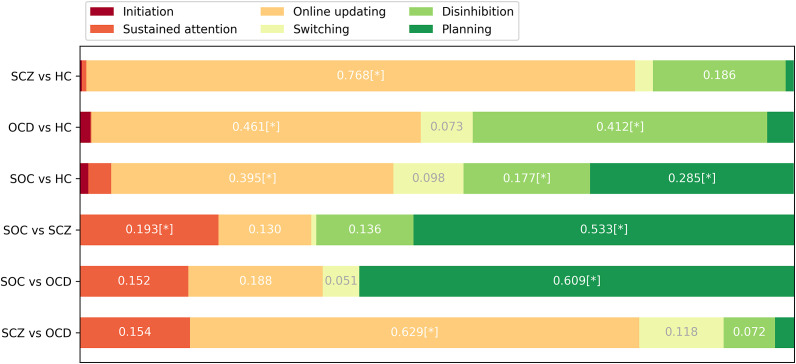
Importance value of six EF components of in each classifier. *represented the core contributors according to importance difference analysis using paired *t*-test.

Figure 3 and Supplementary Table S5 show the values of component importance. Using pair-sample *t*-tests, we found that the importance value of online updating (0.768 ± 0.115) was significantly higher than the other five components (*p* < 0.001) when classifying SCZ patients from HC. In the classification of OCD patients versus HC, both disinhibition (0.412 ± 0.193) and online updating (0.461 ± 0.186) jointly played a decisive role, with no significant difference between them (*p* = 0.923), but significantly higher than those of the other components (*p* < 0.001). For the classification of SOC patients versus HC, several components showed relatively high importance values, including online updating (0.395 ± 0.151), planning (0.285 ± 0.186), and disinhibition (0.177 ± 0.121). These three components were significantly higher than the others, among them, online updating and planning were comparable in importance (*p* = 0.148), while online updating was significantly more important than disinhibition (*p* < 0.001). No significant difference in importance was found between planning and disinhibition (*p* = 0.012).

When classifying SOC patients from OCD patients, planning (0.609 ± 0.171) had the highest importance value, which was significantly higher than the other five components. Although the model’s performance in classifying SOC from SCZ appeared to be less well-fitting than other models, we could still observe the chief EF components contributing to the classification performance. In this ‘compromised’ model, planning (0.533 ± 0.239) still had the highest importance value, and sustained attention (0.193 ± 0.197) also played an important role in the classification model. Finally, online updating (0.629 ± 0.171) was the essential feature in classifying SCZ patients from OCD patients. Results of the paired sample *t*-tests are shown in Supplementary Tables S6–S11.

To account for the potential influence of general intellectual ability on specific cognitive deficits, we conducted additional analysis by incorporating the estimated IQ scores as an input feature in both GBDT and SVM models. Our analyses revealed that incorporating IQ scores provided only marginal improvements to classification performance across most metrics in both models. More importantly, feature importance analysis demonstrated consistently small contributions from IQ scores (importance values ranging from 0.010 to 0.122 across all classification tasks), and the core executive function components identified in our primary analysis remained the most discriminative features even after IQ score inclusion. The details are shown in Supplementary Tables S12 and S13.

## Discussion

While EF impairments in SCZ and OCD are well established, our study provides novel insights into the distinctive neurocognitive architecture of SOC patients. Our main findings supported the existence of EF impairments in patients with SOC, SCZ, and OCD, which can be manifested in differentiating these clinical groups from HC. Furthermore, different components of EF contributed differently to the classification of the three clinical groups. Specifically, online updating served as the definitive feature distinguishing SCZ patients from HC, whereas disinhibition and online updating jointly determined the classification between OCD patients and HC. On the other hand, online updating, planning, and disinhibition collectively served as key distinguishing features to differentiate SOC patients from HC. Moreover, EF distinguished SOC from OCD patients better than SOC from SCZ patients, suggesting that the profile of EF deficits in SOC patients is more analogous to that of SCZ patients.

Compared to HC, patients with SOC, SCZ, and OCD demonstrated varying degrees of executive dysfunction. Specifically, all three clinical groups exhibited significant impairments in online updating and disinhibition; and both SOC and SCZ patients exhibited prominent deficits in sustained attention and planning compared to HC. Further classification results also showed that the model trained on the data with EF could distinguish these three clinical groups from HC. Previous research emphasized that impaired EF could be a shared feature across a wide range of mental disorders, including SCZ and OCD (Bloemen et al., 2018; Roye, Calamia, & Robinson, 2022; Shanmugan et al., 2016), and these two disorders have common neurobiological substrates (Goodkind et al., 2015; Sha, Wager, Mechelli, & He, 2019). These overlapping cognitive and neurobiological alterations may be the underlying cause of SOC (Goodkind et al., 2015; Wang et al., 2020). Moreover, when classifying the clinical groups, the models of SCZ versus OCD, and SOC versus OCD are acceptable, but the model for SOC versus SCZ is suboptimal. These findings indicated that the patterns of executive dysfunction of SOC may be more similar to SCZ.

While executive dysfunction constitutes a shared feature across clinical groups, our novel findings reveal distinct patterns of impairment across specific EF components. Although traditional group comparisons demonstrated that compared to HC, SCZ patients displayed widespread EF impairments, including deficits in initiation, sustained attention, online updating, disinhibition, and planning, the machine learning feature importance analysis highlighted online updating deficits as a uniquely prominent discriminative feature for distinguishing SCZ patients from both HC and OCD patients. This aligned with earlier research highlighting online updating as a potential specific cognitive marker for SCZ patients (Ding, Fu, Luo, & Wu, 2024; Gold & Luck, 2023). Furthermore, when classifying OCD patients from HC, disinhibition and online updating act synergistically as core discriminative features, consistent with prior research highlighting their role in OCD’s pathophysiology (Benzina et al., 2016; Bora, 2020). Notably, compared to HC, SOC patients exhibited even more widespread network-level impairments encompassing not only online updating and disinhibition but also planning, a pattern that reflected the multidimensional cognitive burden of comorbid conditions. This result revealed that SOC patients did not merely present with the overlapping cognitive impairments of SCZ and OCD (e.g., SCZ-related online updating deficits and OCD-linked disinhibition); they also demonstrated additive effects, most prominently in planning dysfunction, which aligned with the ‘double jeopardy’ hypothesis of SOC patients’ pathogenesis (Cunill et al., 2023; Wang et al., 2020).

For planning, the feature importance analysis via the machine learning approach also indicated that the planning component not only serves as a core feature in distinguishing SOC patients from HC but also constitutes an important marker in differentiating SOC patients from SCZ and OCD patients. According to the SAS model, planning depends on the top-down control function, which enables the formulation, organization, and dynamic adjustment of behavioral sequences to achieve a goal (Norman & Shallice, 1986). Its impairment may drive SOC patients’ greater functional difficulties in daily tasks requiring foresight and strategy adjustment, which partially may explain why SOC patients often experience greater functional disability than those with SCZ or OCD alone (Bener et al., 2018). However, it is noteworthy that the machine learning models exhibited limited discriminatory power when differentiating between SOC and SCZ patients. This weak discriminability might suggest that, while SOC and SCZ might differ in the severity of planning impairments, the two groups might share substantial overlap in their broader EF deficit profiles, limiting the models’ ability to distinguish these two clinical groups.

The findings of group comparisons also revealed that both SOC and SCZ patients exhibited significant impairments in sustained attention relative to HC and OCD. Sustained attention, conceptualized as the capacity to maintain focused attention and consistent alertness over prolonged periods, serves as a fundamental cognitive scaffold that underpins individual differences in executive functioning(deBettencourt, Keene, Awh, & Vogel, 2019; Wooten et al., 2024). Hence, the compromised sustained attention observed in these clinical groups may potentially interact with processes such as the online updating and goal-directed planning, and this co-occurrence collectively contributes to the global executive dysfunction observed in these populations.

In contrast to our hypothesis, the clinical groups show no prominent deficits in the switching component, although the inability to switch attention has been regarded as the core feature of OCD. In this study, we used the effective shifting number in MSET as the index of switching, which may not be sensitive enough. More sensitive and specific tests (e.g., the Wisconsin Card Sorting Test) to explore switching in SOC and OCD patients should be used in future research (Nelson, 1976). Notably, a previous review suggested that OCD patients may have intact cognitive flexibility at the behavioral levels while showing abnormalities at the neural level (Gruner & Pittenger, 2017). Such discrepancy regarding cognitive inflexibility may be related to the insufficient sensitivity of the behavioral probes used and potential compensatory processes that OCD patients may adopt (Gruner & Pittenger, 2017). Neuroimaging studies on EF processing in the three clinical groups may be needed in the future.

Notably, our sample size was relatively small (particularly for the SOC group), and this study involved only a single site. These shortcomings undermined the stability and generalizability of the classification models. Although we employed strict cross-validation procedures (50-fold cross-validation) to mitigate the risk of overfitting, and comprehensively evaluated the model performance, our findings gathered in a small sample were likely affected by sampling bias, reducing the robustness of feature selection outcomes. Future multicenter collaboration research is needed to expand the sample size. Such large-scale replications will help validate the robustness of our current findings, and improve the generalizability of the classification model to broader clinical populations.

This study also has other limitations. First, the lack of complete temporal data regarding symptom onset in the SOC group limited our ability to infer any causal relationship between OCD and SCZ pathophysiology (e.g., whether OCD is a risk factor for SCZ, a secondary manifestation of SCZ pathology, or an independent comorbidity). Additionally, despite using concurrent DSM-IV diagnoses of schizophrenia and OCD as the sole SOC inclusion criterion, potential diagnostic misclassification is common in cross-sectional studies. Future longitudinal studies are needed to track the dynamic emergence of obsessive-compulsive symptoms, which will help clarify the temporal sequence and refine classification of the SOC patients. Second, we did not measure the obsessive-compulsive symptoms of SCZ nor the psychotic symptoms in OCD, which precluded the possibility of exploring the potential relationship between executive dysfunction and clinical symptoms using a transdiagnostic sample. Third, although we only captured six specific components of EF based on the SAS model, we did not include other components, such as fluency. Fourth, cognitive performance is highly sensitive to psychotropic exposure (Chandramouleeshwaran, Khan, Inglis, & Rajji, 2024). Although we included antipsychotic dosage and antidepressant drug-class as covariates, we did not account for more granular medication variables, such as treatment duration. Finally, in this study, the EF was considered the ‘cold’ cognition (emotion-independent and logically-based), but awe did not assess participants’ ‘hot’ cognition (which involves processing stimuli with emotional components) (Salehinejad, Ghanavati, Rashid, & Nitsche, 2021). Future studies should focus on the specific features of ‘hot’ cognition on SOC, such as motivation and emotion.

To conclude, our findings supported that executive dysfunctions were prevalent among SOC, SCZ, and OCD patients, but these three clinical groups differed in component-specific EF impairments. Moreover, obsessive-compulsive symptoms could exacerbate the EF impairments in SCZ patients. These findings may enable the identification of specific cognitive signatures for distinct clinical phenotypes and provide a neurocognitive framework for understanding SOC pathophysiology, potentially improving differential diagnosis and treatment targeting for these complex psychiatric conditions.

## Supporting information

10.1017/S0033291726104061.sm001 Chu et al. supplementary material Chu et al. supplementary material

## Supplementary Material

The supplementary material for this article can be found at http://doi.org/10.1017/S0033291726104061.
