# Supplementary material for: Characterizing executive dysfunctions in patients with schizo-obsessive comorbidity: comparing schizophrenia with obsessive-compulsive disorder
Source: Psychol Med. 2026 Apr 27;56:e116. doi: 10.1017/S0033291726104061 (PMC13125927; doi:10.1017/S0033291726104061)
Supplement: Chu et al. supplementary material [file S0033291726104061sup001.docx]

**Supplementary Materials**

**Usage of medication**

**Details of machine learning method**

**Table S1 The** **group differences** **in the raw scores of the ten** **parameters of executive function test**

**Table S2. Group Comparison of specific components of executive function**

**Table S3 Spearman correlations between the executive function components and PANSS in SOC and SCZ groups**

**Table S4. Classification results**

**Table S5 The feature importance of GBDT classifiers**

**Table S6 The difference of importance value between SCZ and HC**

**Table S7 The difference of importance value between OCD and HC**

**Table S8 The difference of importance value between SOC and HC**

**Table S9 The difference of importance value between SCZ and SOC**

**Table S10 The difference of importance value between SOC and OCD**

**Table S11 The difference of importance value between SCZ and OCD**

**Table S12. Classification results after putting the estimated IQ scores into model**

**Table S13. The feature importance of GBDT classifiers after putting the estimated IQ scores into model**

**Usage of medication**

Out of 175 participants, there were 28 unmedicated patients, with3 SCZ, 18 OCD, and 6 SOC. The antipsychotic dose was transformed into the olanzapine-equivalent dose based on defined daily doses (DDDs) presented by the WHO Collaborative Center for Drug Statistics Methodology (Leucht et al., 2016). The SOC (Mean±SD: 16.27±9 9.14) group received signiﬁcantly higher mean doses of antipsychotic medications than the SCZ group (Mean±SD:11.72±7.05, t=2.670, p=0.009). Moreover, there were also 15 OCD patients who received low-dose antipsychotic medications (Mean±SD:3.74±3.57). The usage of antidepressant drugs was presented as follows：

|  | SCZ | OCD | SOC |
| --- | --- | --- | --- |
| SSRI only | 13 | 35 | 16 |
| SNRI only | 1 | 1 | 0 |
| TCA only | 1 | 0 | 2 |
| Other atypical antidepressants | 5 | 2 | 1 |
| Combining with two kinds of antidepressant drugs | 1 | 14 | 4 |

SSRI: selective serotonin reuptake inhibitors; SNRI: Serotonin-Norepinephrine Reuptake Inhibitor; TCA: Tricyclic Antidepressants.

- Leucht, S., Samara, M., Heres, S., & Davis, J. M. (2016). Dose Equivalents for Antipsychotic Drugs: The DDD Method. Schizophrenia bulletin, 42 Suppl 1(Suppl 1), S90–S94. https://doi.org/10.1093/schbul/sbv167

**Details of** **machine learning method**

- **Pair Preparation Details**

Although trained on the same EF components, the models could still have different classification capacities for different groups. Thus, we organized EF components data to form different classification tasks. Specifically, we selected two of the four groups (HC, SCZ, OCD, and SOC) as a pair, and then merged and shuffled the pair’s samples as a dataset for model training and testing. For example, when HC and SCZ were selected as a pair, the models (i.e., SVM and GBDT) were trained on their merged and shuffled training data. Then, two classifiers (i.e., one for SVM and one for GBDT) were obtained to classify HC and SCZ. Thus, we generated six pairs, i.e., HC vs. SCZ, HC vs. OCD, HC vs. SOC, SCZ vs. OCD, SCZ vs. SOC, and OCD vs. SOC. Each pair had a dataset and two independent classifiers based on SVM and GBDT.

- **Parameter Tuning Details in Model Training**

Most SVM and GBDT model parameters were set to the default values in the scikit-learn package. Only the “C=0.5” and “kernel=linear” of SVM and “n_estimators=10”, “learning_rate=0.1”, and “max_depth=1” of GBDT were tuned according to the performance. Specifically, “C” is a regularization parameter, “kernel” specifies the kernel type used in the model, “max_depth” limits the number of nodes in the tree, “n_estimators” is the number of boosting stages to perform, and “learning_rate” shrinks the contribution of each tree. There is a trade-off between “learning_rate” and “n_estimators”.

- **Brief introduction of AUC, Precision, Recall, and F1 in Model Test**

AUC, Precision, Recall, and F1 are all popular metrics to evaluate classification performance, where Precision evaluates how many true positive samples are over all samples classified as positive ones; Recall evaluates how many positive samples are correctly classified over all positive ones; F1 is equal to 2*Precision*Recall/(Precision+Recall). AUC is a more comprehensive metric, which computes the area under the curve using the trapezoidal rule. It is not sensitive to the bias between positive and negative samples. Notably, we calculate the value of these metrics for each label, i.e., each label will be viewed as a positive sample, and then treat the average value as the final performance. In machine learning domains, no explicit threshold of these metrics to indicate whether the performance of classifiers is good enough. The higher the value of these metrics, the better the classifiers. Thus, the metrics are usually utilized to compare the performance between classifiers. However, empirically, when the value of AUC, Precision, Recall, and F1 is larger than 0.6, it means the classifiers work; when the value is larger than 0.75, the performance is satisfactory (<https://scikit-learn.org/stable/api/sklearn.metrics.html>)

- **Details of feature Importance of GBDT**

The GBDT could estimate the six components’ importance via their classification contributions. Each component had 50 importance values for one pair, as we had 50 independent experiments. Then, the importance difference between components was estimated using a paired sample t-test, according to their paired data. Considering that six components were compared by paired sample t-tests, which would involve (6*6 - 6)/2 = 15 individual t-tests, the significance level was set as 0.003(0.05/15) after correction for multiple comparisons.

**Table S1. The group differences in the raw scores of the ten parameters of executive function test**

| **Components** | **Parameters** | **SCZ**  **(n=68)** | **OCD**  **(n=70)** | **SOC**  **(n=37)** | **HC**  **(n=59)** | **F** | **p** | ***Eta^2^*** | ***Post-hoc*** |
| --- | --- | --- | --- | --- | --- | --- | --- | --- | --- |
| **Initiation** | HSCT-A (correct responses) | 14.5±1.56 | 14.8±0.47 | 14.57±0.8 | 14.95±0.29 | 2.859 | 0.038 | 0.036 | 1<4 |
| **Sustained attention** | SART (correct responses) | 0.96±0.06 | 0.97±0.04 | 0.94±0.06 | 0.98±0.02 | 7.761 | <0.001 | 0.092 | 1,3<2,4 |
| **Online updating** | LNST (total passed) | 12.97±4.15 | 16.13±4.08 | 13.14±4.19 | 17.61±4.43 | 16.896 | <0.001 | 0.181 | 1,3<2,4 |
|  | n-back(2-back correct) | 0.37±0.19 | 0.4±0.22 | 0.34±0.19 | 0.53±0.23 | 8.633 | <0.001 | 0.101 | 1,2,3<4 |
| **Switching** | SET correct shift time | 12.53±18.07 | 13.91±18.91 | 8.16±12.68 | 17.05±25.89 | 1.574 | 0.197 | 0.02 |  |
| **Disinhibition** | SART (commission errors) | 0.54±0.26 | 0.54±0.26 | 0.54±0.24 | 0.56±0.25 | 0.074 | 0.974 | 0.001 |  |
|  | HSC-B (total errors) | 7.74±4.05 | 6.73±4.08 | 8.11±3.83 | 3.73±2.84 | 15.524 | <0.001 | 0.168 | 1,2,3>4 |
|  | SET (rule-breaking errors) | 1.09±5.75 | 0.9±3.6 | 0.97±1.76 | 0.47±1.71 | 0.292 | 0.831 | 0.004 |  |
| **Planning** | SET (total profile score) | 2.91±1.05 | 3.23±0.82 | 2.41±0.96 | 3.54±0.75 | 13.612 | <0.001 | 0.151 | 3<1<2<4 |
|  | SET(raw score) | 4.56±1.39 | 5.1±1.14 | 3.97±1.36 | 5.37±1.07 | 11.912 | <0.001 | 0.134 | 3<1<2,4 |

Note: HSCT: Hayling Sentence Completion Test, SET: Six Elements Test, SART: Sustained Attention to Response Task, LNST: Letter-Number Span test;

**Table S2.** **Group Comparison of specific components of executive function**

|  | **1.SCZ**  **(n=68)** | **2.OCD**  **(n=70)** | **3.SOC**  **(n=37)** | **4.HC**  **(n=59)** | **F** | **p** | **Eta^2^** | **post-hoc^a^** |  |
| --- | --- | --- | --- | --- | --- | --- | --- | --- | --- |
| **I****nitiation** | -1.55±5.4 | -0.52±1.62 | -1.32±2.77 | 0±1 | 2.859 | 0.038 | 0.036 | 1<4 | |
| **Sustained attention** | -1.08±2.33 | -0.35±1.83 | -1.8±2.66 | 0±1 | 7.761 | <0.001 | 0.092 | 1,3<2,4 | |
| **Online updating** | -0.88±0.69 | -0.46±0.78 | -0.92±0.78 | 0±0.8 | 18.047 | <0.001 | 0.191 | 1,3<2<4 | |
| **Switching** | -0.17±0.7 | -0.12±0.73 | -0.34±0.49 | 0±1 | 1.574 | 0.197 | 0.020 |  | |
| **Disinhibition** | 0.57±1.23 | 0.41±0.89 | 0.58±0.69 | 0±0.6 | 4.931 | 0.002 | 0.060 | 1,2,3>4 | |
| **Planning** | -0.8±1.32 | -0.34±1.06 | -1.41±1.21 | 0±0.98 | 13.428 | <0.001 | 0.149 | 3<1<2, 4 | |

Note: SCZ: Schizophrenia; OCD: Obsessive-compulsive disorder; SOC: Schizo-obsessive comorbidity; HC: Healthy controls; the numbers in the post hoc-column refer to 1: SCZ group, 2: OCD group;3 SOC group, 4: HC group; a: the numbers in the post hoc-column refer to 1: patients with Schizophrenia, 2: patients with obsessive-compulsive disorder group;3 patients with Schizo-obsessive comorbidity, 4: Healthy controls.

**Table S3 Spearman correlations between the executive function components and clinical symptoms in clinical groups**

|  |  | **initiation** | **Sustained attention** | **Online**  **updating** | **Switching** | **Disinhibition** | **Planning** |
| --- | --- | --- | --- | --- | --- | --- | --- |
| **SCZ** | **PANSS_P** | -0.19(0.12) | -0.004(0.972) | -0.199(0.104) | 0.113(0.36) | 0.192(0.116) | 0.082(0.507) |
|  | **PANSS_N** | -0.256(0.035) | -0.224(0.066) | -0.027(0.828) | -0.042(0.735) | 0.171(0.164) | -0.247(0.042) |
|  | **PANSS_G** | -0.21(0.086) | -0.016(0.897) | -0.028(0.821) | 0.167(0.173) | 0.117(0.343) | 0.013(0.915) |
|  | **BDI** | **-0.426**(<0.001)** | -0.126(0.322) | -0.024(0.853) | -0.04(0.753) | 0.016(0.902) | -0.028(0.828) |
|  | **BAI** | -0.125(0.313) | -0.029(0.815) | -0.046(0.712) | 0.125(0.315) | -0.054(0.667) | -0.014(0.908) |
| **OCD** | **Obsession** | 0.011(0.93) | -0.031(0.797) | -0.108(0.373) | 0.158(0.191) | -0.196(0.104) | 0.018(0.884) |
|  | **Compulsion** | 0.027(0.824) | -0.137(0.259) | 0.093(0.444) | 0.133(0.273) | 0.114(0.349) | -0.013(0.917) |
|  | **BDI** | -0.168(0.167) | -0.062(0.612) | 0.203(0.094) | 0.04(0.743) | -0.102(0.403) | -0.011(0.931) |
|  | **BAI** | -0.179(0.139) | -0.216(0.073) | 0.067(0.582) | -0.011(0.926) | -0.056(0.646) | 0.044(0.719) |
| **SOC** | **PANSS_P** | -0.091(0.596) | -0.263(0.122) | -0.383(0.021) | 0.106(0.539) | 0.183(0.285) | -0.097(0.573) |
|  | **PANSS_N** | -0.403(0.015) | -0.287(0.09) | -0.451 (0.006) | -0.22(0.197) | **0.541**(<0.001)** | -0.258(0.129) |
|  | **PANSS_G** | -0.371(0.026) | -0.172(0.315) | -0.358(0.032) | -0.011(0.95) | 0.404(0.014) | -0.149(0.386) |
|  | **Obsession** | -0.254(0.129) | -0.085(0.616) | 0.083(0.625) | 0.144(0.396) | 0.15(0.377) | 0.069(0.684) |
|  | **Compulsion** | -0.286(0.086) | -.345(0.037) | -0.136(0.423) | 0.252(0.132) | 0.208(0.216) | -0.162(0.339) |
|  | **BDI** | -0.017(0.922) | -0.124(0.471) | -0.128(0.457) | 0.272(0.109) | 0.071(0.68) | 0.062(0.72) |
|  | **BAI** | 0.153(0.374) | -0.228(0.18) | -0.163(0.343) | 0.237(0.163) | 0.152(0.375) | 0.11(0.521) |

Note: values in table represent: t value (p value), ** indicates statistical significance that remained after Bonferroni correction. Specifically, for SCZ patients, the significance threshold was set at p < 0.0016 (calculated as 0.05/(5×6)); for OCD patients, the threshold was p < 0.0021 (0.05/(4×6)); and for SOC patients, the threshold was p < 0.0012 (0.05/(7×6)).

**Table S4. Classification results**

| **Classifier** | **Classification** | **AUC** | **Precision** | **Recall** | **F1** |
| --- | --- | --- | --- | --- | --- |
| **GBDT** | **SCZ vs HC** | 0.799±0.071 | 0.724±0.078 | 0.708±0.080 | 0.706±0.081 |
|  | **OCD vs HC** | 0.657±0.096 | 0.615±0.119 | 0.588±0.104 | 0.571±0.129 |
|  | **SOC vs HC** | 0.825±0.087 | 0.793±0.078 | 0.742±0.092 | 0.722±0.106 |
|  | **SOC vs SCZ** | 0.575±0.103 | 0.453±0.162 | 0.635±0.090 | 0.509±0.120 |
|  | **SOC vs OCD** | 0.755±0.104 | 0.751±0.126 | 0.714±0.110 | 0.676±0.136 |
|  | **SCZ vs OCD** | 0.628±0.095 | 0.640±0.092 | 0.611±0.095 | 0.607±0.099 |
| **SVM** | **SCZ vs HC** | 0.790±0.088 | 0.743±0.086 | 0.713±0.083 | 0.713±0.084 |
|  | **OCD vs HC** | 0.663±0.107 | 0.639±0.103 | 0.608±0.106 | 0.604±0.107 |
|  | **SOC vs HC** | 0.863±0.070 | 0.773±0.082 | 0.749±0.082 | 0.746±0.085 |
|  | **SOC vs SCZ** | 0.487±0.135 | 0.441±0.134 | 0.602±0.098 | 0.498±0.110 |
|  | **SOC vs OCD** | 0.740±0.111 | 0.730±0.110 | 0.713±0.104 | 0.707±0.108 |
|  | **SCZ vs OCD** | 0.599±0.096 | 0.587±0.089 | 0.559±0.077 | 0.549±0.078 |

Note: SCZ: Schizophrenia; OCD: Obsessive-compulsive disorder; SOC: Schizo-obsessive comorbidity; HC: Healthy controls; GBDT: Gradient Boosting Decision Tree; SVM: Support Vector Machine; the values in the table were represented to the mean±SD;

**Table S5 The feature importance of GBDT classifiers**

|  | Initiation | Sustained  attention | Online  updating | Switching | Disinhibition | Planning |
| --- | --- | --- | --- | --- | --- | --- |
| SCZ vs HC | 0.003±0.016 | 0.006±0.018 | 0.768±0.115 | 0.025±0.040 | 0.186±0.108 | 0.011±0.027 |
| OCD vs HC | 0.015±0.037 | 0.001±0.009 | 0.461±0.186 | 0.073±0.079 | 0.412±0.193 | 0.037±0.061 |
| SOC vs HC | 0.012±0.037 | 0.032±0.049 | 0.395±0.151 | 0.098±0.112 | 0.177±0.121 | 0.285±0.186 |
| SOC vs SCZ | 0.001±0.008 | 0.193±0.197 | 0.130±0.132 | 0.007±0.022 | 0.136±0.177 | 0.533±0.239 |
| SOC vs OCD | 0.000±0.000 | 0.152±0.132 | 0.188±0.136 | 0.051±0.078 | 0.000±0.000 | 0.609±0.171 |
| SCZ vs OCD | 0.000±0.000 | 0.154±0.151 | 0.629±0.171 | 0.118±0.125 | 0.072±0.114 | 0.027±0.054 |

Note: SCZ: Schizophrenia; OCD: Obsessive-compulsive disorder; SOC: Schizo-obsessive comorbidity; HC: Healthy controls; the values in the table were represented as the mean±SD;

**Table S6. The difference of importance value between SCZ and HC**

| **SCZ vs HC** | **Initiation** | **Sustained**  **attention** | **Online updating** | **Switching** | **Disinhibition** | **Planning** |
| --- | --- | --- | --- | --- | --- | --- |
| **Initiation** |  |  |  |  |  |  |
| **Sustained**  **attention** | -1.662(0.103) |  |  |  |  |  |
| **Online updating** | -34.525(<0.001) | -34.401(<0.001) |  |  |  |  |
| **Switching** | -3.800(<0.001) | -2.437(0.018) | 32.396(<0.001) |  |  |  |
| **Disinhibition** | -9.352(<0.001) | -8.925(<0.001) | 13.899(<0.001) | -7.733(<0.001) |  |  |
| **Planning** | -2.457(0.018) | -1.137(0.261) | 34.082(<0.001) | 1.265(0.212) | 8.207(<0.001) |  |

Note: values in table represent: t value (p value), significance level: p<0.003;

**Table S7. The difference of importance value between OCD and HC**

| **OCD vs HC** | **Initiation** | **Sustained**  **attention** | **Online updating** | **Switching** | **Disinhibition** | **Planning** |
| --- | --- | --- | --- | --- | --- | --- |
| **Initiation** |  |  |  |  |  |  |
| **Sustained**  **attention** | 1.227(0.226) |  |  |  |  |  |
| **Online updating** | -17.385(<0.001) | -17.996(<0.001) |  |  |  |  |
| **Switching** | -3.521(0.001) | -4.608(<0.001) | 15.762(<0.001) |  |  |  |
| **Disinhibition** | -14.761(<0.001) | -16.233(<0.001) | -0.098(0.923) | -12.895(<0.001) |  |  |
| **Planning** | -1.948(0.057) | -3.385(0.001) | 15.770(<0.001) | 1.035(0.306) | 13.844(<0.001) |  |

Note: values in table represent: t value (p value), significance level: p<0.003;

**Table S8.** **The difference of importance value between SOC and HC**

| **SOC vs HC** | **Initiation** | **Sustained**  **attention** | **Online updating** | **Switching** | **Disinhibition** | **Planning** |
| --- | --- | --- | --- | --- | --- | --- |
| **Initiation** |  |  |  |  |  |  |
| **Sustained**  **attention** | -2.950(0.005) |  |  |  |  |  |
| **Online updating** | -12.776(<0.001) | -10.400(<0.001) |  |  |  |  |
| **Switching** | -4.663(<0.001) | -2.516(0.015) | 7.774(<0.001) |  |  |  |
| **Disinhibition** | -10.677(<0.001) | -7.552(<0.001) | 4.717(<0.001) | -4.305(<0.001) |  |  |
| **Planning** | -9.678(<0.001) | -7.797(<0.001) | 1.470(0.148) | -5.548(<0.001) | -2.607(0.012) |  |

Note: values in table represent: t value (p value), significance level: p<0.003;

**Table S9. The difference of importance value between SCZ and SOC**

| **SCZ vs SOC** | **Initiation** | **Sustained**  **attention** | **Online updating** | **Switching** | **Disinhibition** | **Planning** |
| --- | --- | --- | --- | --- | --- | --- |
| **Initiation** |  |  |  |  |  |  |
| **Sustained**  **attention** | -7.918(<0.001) |  |  |  |  |  |
| **Online updating** | -6.577(<0.001) | 2.678(0.010) |  |  |  |  |
| **Switching** | -1.641(0.107) | 7.494(<0.001) | -8.060(<0.001) |  |  |  |
| **Disinhibition** | -5.917(<0.001) | 7.494(<0.001) | 5.914(<0.001) | -12.025(<0.001) |  |  |
| **Planning** | -17.623(<0.001) | 0.765(0.448) | -1.228(0.225) | -5.473(<0.001) | -17.217(<0.001) |  |

Note: values in table represent: t value (p value), significance level: p<0.003;

**Table S10. The difference of importance value between SOC and OCD**

| **OCD vs SOC** | **Initiation** | **Sustained**  **attention** | **Online updating** | **Switching** | **Disinhibition** | **Planning** |
| --- | --- | --- | --- | --- | --- | --- |
| **Initiation** |  |  |  |  |  |  |
| **Sustained**  **attention** | -10.262(<0.001) |  |  |  |  |  |
| **Online updating** | -7.507(<0.001) | 1.156(0.253) |  |  |  |  |
| **Switching** | -3.766(<0.001) | 4.831(<0.001) | 3.501(0.001) |  |  |  |
| **Disinhibition** | -1.000(0.322) | 9.556(<0.001) | 7.156(<0.001) | 3.460(0.001) |  |  |
| **Planning** | -24.112(<0.001) | -13.341(<0.001) | -12.640(<0.001) | -14.886(<0.001) | -24.040(<0.001) |  |

Note: values in table represent: t value ( p value), significance level: p<0.003;

**Table S11. The difference of importance value between SCZ and OCD**

| **SCZ vs OCD** | **Initiation** | **Sustained**  **attention** | **Online updating** | **Switching** | **Disinhibition** | **Planning** |
| --- | --- | --- | --- | --- | --- | --- |
| **Initiation** |  |  |  |  |  |  |
| **Sustained**  **attention** | -6.377(<0.001) |  |  |  |  |  |
| **Online updating** | -20.022(<0.001) | -9.534(<0.001) |  |  |  |  |
| **Switching** | -5.641(<0.001) | 2.320(0.025) | 14.863(<0.001) |  |  |  |
| **Disinhibition** | -3.428(0.001) | 2.752(0.008) | 13.140(<0.001) | 0.641(0.525) |  |  |
| **Planning** | -2.108(0.040) | 4.509(<0.001) | 18.425(<0.001) | 3.090(0.003) | 1.889(0.065) |  |

Note: values in table represent: t value (p value), significance level: p<0.003;

**Table S12. Classification results after putting the estimated IQ scores into model**

| **Classifier** | **Classification** | **AUC** | **Precision** | **Recall** | **F1** |
| --- | --- | --- | --- | --- | --- |
| **GBDT** | **SCZ vs HC** | 0.842±0.066 | 0.746±0.080 | 0.766±0.077 | 0.746±0.080 |
|  | **OCD vs HC** | 0.650±0.097 | 0.580±0.113 | 0.612±0.130 | 0.580±0.113 |
|  | **SOC vs HC** | 0.801±0.099 | 0.741±0.097 | 0.766±0.101 | 0.741±0.097 |
|  | **SOC vs SCZ** | 0.577±0.111 | 0.636±0.089 | 0.480±0.166 | 0.636±0.089 |
|  | **SOC vs OCD** | 0.753±0.110 | 0.725±0.092 | 0.744±0.105 | 0.725±0.092 |
|  | **SCZ vs OCD** | 0.607±0.089 | 0.595±0.088 | 0.612±0.087 | 0.595±0.088 |
| **SVM** | **SCZ vs HC** | 0.783±0.073 | 0.721±0.079 | 0.744±0.068 | 0.721±0.079 |
|  | **OCD vs HC** | 0.657±0.087 | 0.625±0.083 | 0.649±0.084 | 0.625±0.083 |
|  | **SOC vs HC** | 0.829±0.087 | 0.739±0.095 | 0.761±0.098 | 0.739±0.095 |
|  | **SOC vs SCZ** | 0.469±0.121 | 0.598±0.082 | 0.424±0.114 | 0.598±0.082 |
|  | **SOC vs OCD** | 0.779±0.097 | 0.751±0.084 | 0.768±0.085 | 0.751±0.084 |
|  | **SCZ vs OCD** | 0.589±0.091 | 0.549±0.072 | 0.577±0.086 | 0.549±0.072 |

**Table S13. The feature importance of GBDT classifiers after putting the estimated IQ scores into model**

|  | **Initiation** | **Sustained attention** | **Online updating** | **Switching** | **Disinhibition** | **Planning** | **IQ** |
| --- | --- | --- | --- | --- | --- | --- | --- |
| **SCZ vs HC** | 0.000±0.000 | 0.005±0.019 | 0.698±0.143 | 0.019±0.036 | 0.178±0.135 | 0.010±0.030 | 0.090±0.081 |
| **OCD vs HC** | 0.007±0.042 | 0.000±0.000 | 0.443±0.174 | 0.040±0.061 | 0.448±0.195 | 0.027±0.057 | 0.035±0.066 |
| **SOC vs HC** | 0.005±0.024 | 0.037±0.075 | 0.370±0.202 | 0.087±0.118 | 0.195±0.124 | 0.294±0.207 | 0.010±0.035 |
| **SOC vs SCZ** | 0.000±0.000 | 0.161±0.144 | 0.097±0.105 | 0.007±0.028 | 0.136±0.163 | 0.511±0.205 | 0.087±0.107 |
| **SOC vs OCD** | 0.000±0.000 | 0.165±0.114 | 0.133±0.125 | 0.058±0.109 | 0.003±0.021 | 0.631±0.185 | 0.010±0.030 |
| **SCZ vs OCD** | 0.000±0.000 | 0.142±0.157 | 0.575±0.203 | 0.078±0.097 | 0.062±0.128 | 0.021±0.072 | 0.122±0.136 |
